# Jellyfish Envenomation Resulting In Vascular Insufficiency And Neurogenic Injury of Upper Limb

**DOI:** 10.5704/MOJ.1511.007

**Published:** 2015-11

**Authors:** CYL Choong, HZ Chan, NA Faruk, KC Bea, O Zulkiflee

**Affiliations:** Department of Orthopaedics & Traumatology, Penang General Hospital, Georgetown, Malaysia

**Keywords:** Jellyfish, Envenomation, Irukandji Syndrome, Marine Injuries

## Abstract

Following a week after a jellyfish sting, a young man presented with regional cyanosis and threat of distal gangrene secondary to vascular spasm in the forearm. The patient also suffered from transient paresis and numbness of the affected upper limb. Contrasted imaging revealed unopacified vessels in the distal forearm and worsening swelling warranted emergency surgical fasciotomy for impending compartment syndrome. This case highlights the occurrence of jellyfish envenomation and the need for early treatment.

## Introduction

Jellyfish envenomation is a common cause of marine injuries worldwide. Although majority of jellyfish stings are benign, there are venomous species that constantly account for fatalities and severe morbidity. Lippmann J and Fenner PJ reported several cases of deadly jellyfish stings in the coastal waters of Peninsular Malaysia, resulting in severe Irukandji-like syndrome that causes profound anaphylactic shock and mortality.

## Case Report

A 21-year old Cambodian presented with one week history of diffuse redness and swelling over his right forearm associated with blackish discolouration over the fingers, after bathing in the sea off Batu Ferringhi beach. He described feeling a sharp burning pain over his right upper arm while swimming, and noticed linear vesiculourticaric lesions over his forearm and upper arm upon immediately escaping out of the water. After a few minutes, he started to experience Irukandji-like symptoms with excessive sweating, tachycardia and generalized muscle aching. Patient was brought to a nearby clinic on the same day where he was prescribed with intramuscular antihistamine and oral antibiotics. However, the pain and swelling of right upper limb worsened over the next few days, with resulting distal cyanosis of the fingers.

**Fig. 1a & 1b:**
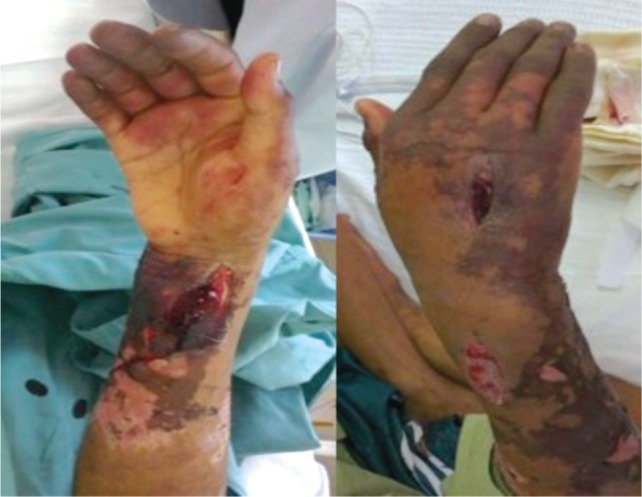
Skin necrosis, thrombophlebitis with impending digital gangrene on admission.

Initial physical examination revealed erythematous plaques with skin necrosis and blistering over the right upper limb mainly over the distal third of forearm. Right middle, ring and little fingers appeared dusky and insensate. (Figure 1a-b) Capillary refill time was more than 2 seconds and oxygen saturation was undetectable by pulse oxymetry. Movement of the small joints were impaired. The rest of physical examination was normal. Plain radiographs were normal. CT angiogram of right upper limb (Figure 2a) showed that superficial and deep palmar arches were not opacified by contrast, suggesting possibility of vasospasm or acute thrombosis.

**Fig. 2a:**
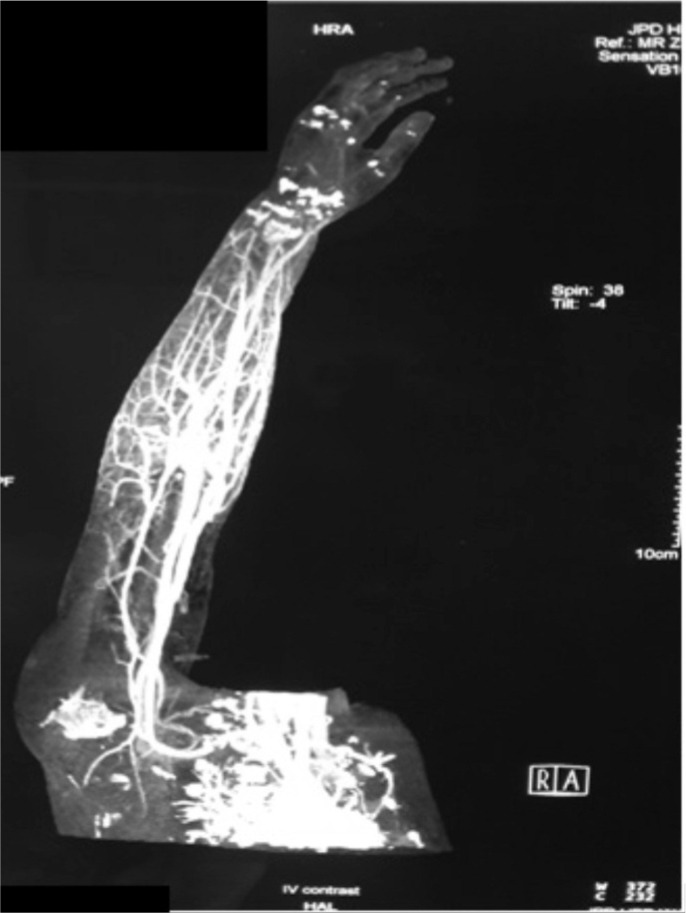
CT Angiogram right upper limb.

Surgical fasciotomy and exploration was done owing to the increasing swelling. Intraoperatively, the surgeon noted presence of serous fluid alone, with no signs of infection. A combination of antithrombotic, antibiotics, and antihistamine therapy was commenced in the ward resulting in recirculation of the digits, resolution of finger numbness and weakness (Figure 3a and b). Repeated CTA right upper limb at 6 weeks after the incident shows recirculation over the distal forearm and palmar arches (Figure 2b).

**Fig. 2b:**
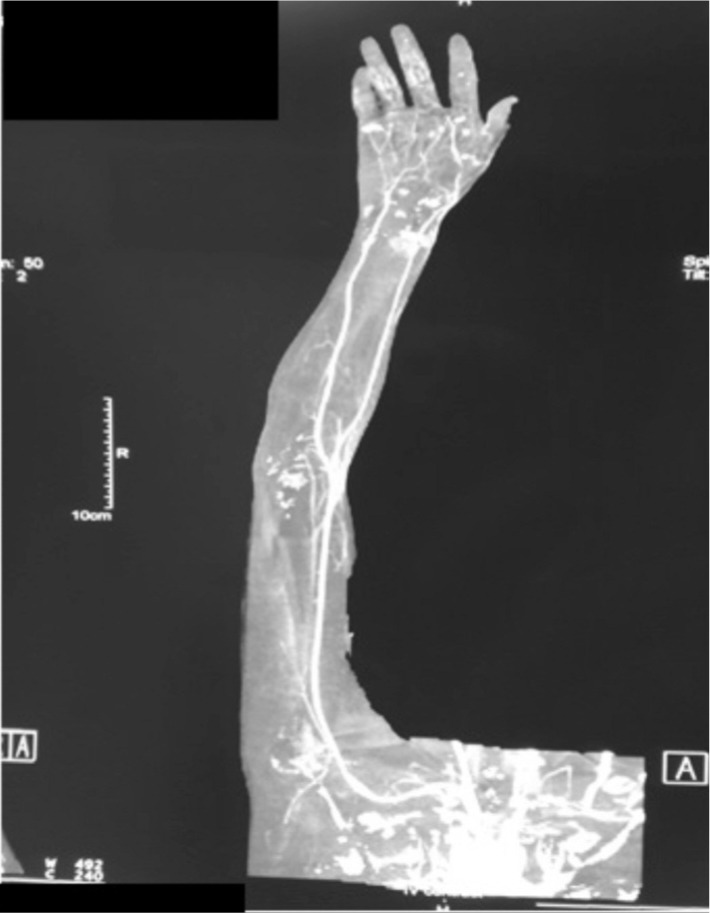
CTA right upper limb (at 6 weeks after jellyfish sting)

**Fig. 3a & 3b:**
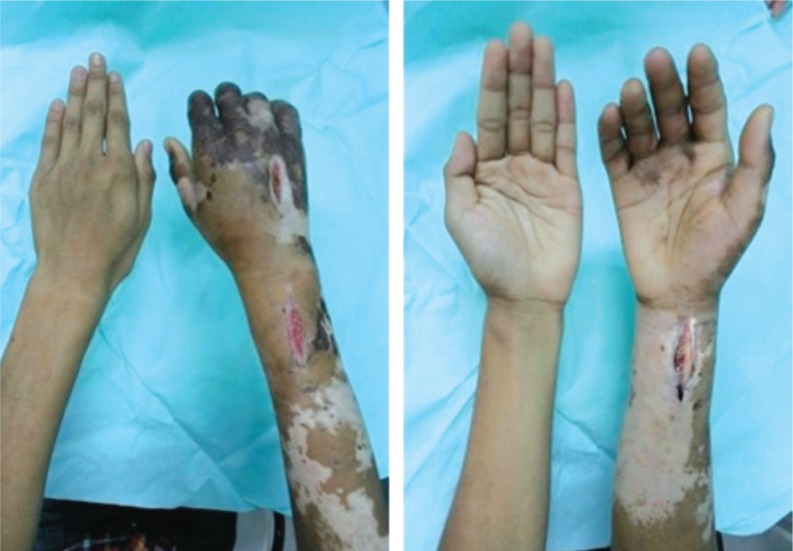
Resolution of thrombophlebitis and digital recirculation with residual hyperpigmentation after 6 weeks.

## Discussion

Jellyfish are marine invertebrates which belong to phylum cnidarian or coelenterates. Presence of millions of nematocysts (or stinging cells) on their tentacles, enables jellyfish to discharge venom when activated by pressure. These nematocyst structures can eject their threads with an approximate force of 0.9 – 2.3kg/cm^2^ thereby inflicting harm to the skin of any close-contact predator^4^.

There are several classes of jellyfish that pose a threat to humans. They are the Chironex fleckeri (box jellyfish or sea wasp), Carukia barnesi, Chrysaora (sea nettle), and Physalia physalis (Portuguese man of war). The Chironex fleckeri is the deadliest of all jellyfish known to mankind with the most rapidly acting venom.

Jellyfish venom is believed to have lipolytic and proteolytic effect on skin and underlying soft tissue including the neurovascular bundle. Local soft tissue oedema produced as a result of the body’s reaction to venom, can compress onto peripheral nerves and vessels. Overtime, it can worsen to resemble compartment syndrome.

Neurotoxic properties of the venom of certain species can cause transient peripheral and autonomic nerves dysfunction which normally resolve spontaneously after a few weeks.

Vascular reactions such as ischemia from vasospasm and thrombophlebitis of the underlying vessels have also been reported^3^. Other rarer reported reactions include angioedema, contact dermatitis, mononeuritis multiplex and cardiopulmonary decompensation^3,4^.

## Conclusion

Jellyfish envenomation is common in the coastal areas of Malaysia. It is a potential life-threatening medical emergency. The public lack of knowledge in this area is because of under reporting of cases. As these cases occur sporadically, it makes trials of first aid and medical treatment even more difficult. The public need to be educated about the risk of jellyfish envenomation and prehospital care in the future to prevent unnecessary delay of treatment, as early treatment may prevent further morbidity.

## References

[1] Lippmann JM, Fenner PJ, Winkel K, Gershwin LA (2011). Fatal and severe box jellyfish stings, including Irukandji stings, in Malaysia, 2000-2010. J Travel Med.

[2] Williamson JP, Fenner J, Burnett J, Rifkin (1996). Venomous And Poisonous Marine Animals:. A Medical And Biological Handbook.

[3] Williamson JA, Burnett JW, Fenner PJ, Hach WunderleV, Hoe LY, Adiga KM (1988). Acute regional vascular insufficiency after jellyfish envenomation. Med J Aust.

[4] Burnett JW, Williamson JA, Fenner PJ (1994). Mononeuritis multiplex after coelenterate sting. Med J Aust.

[5] Fenner PJ, Williamson JA (1996). Worldwide deaths and severe envenomation from jellyfish stings. Med J Aust.

